# Functional enrichment analysis of mutated genes in children with hyperthyroidism

**DOI:** 10.3389/fendo.2023.1213465

**Published:** 2023-10-09

**Authors:** Xiaojian Mao, Liangliang Tang, Hongyi Li, Wen Zhang, Li Liu, Heyong Wang, Abdalbari Headar

**Affiliations:** ^1^ Department of Genetics and Endocrinology, Guangzhou Women and Children's Medical Center, Guangzhou Medical University, Guangdong Provincial Clinical Research Center for Child Health, Guangzhou, China; ^2^ Center of Big Data and Business Itelligent, South China University of Technology, Guangzhou, China

**Keywords:** hyperthyroidism, bioinformatics, gene mutation, enrichment analysis, gene ontology enrichment

## Abstract

**Objective:**

Hyperthyroidism in Chinese children is relatively high and has been increasing in recent years, which has a significant impact on their healthy development. Hyperthyroidism is a polygenic disorder that presents greater challenges in terms of prediction and treatment than monogenic diseases. This study aims to elucidate the associated functions and gene sets of mutated genes in children with hyperthyroidism in terms of the gene ontology through GO enrichment analysis and in terms of biological signaling pathways through KEGG enrichment analysis, thereby enhancing our understanding of the expected effects of multiple mutated genes on hyperthyroidism in children.

**Methods:**

Whole-exome sequencing was performed on the DNA samples of children with hyperthyroidism. Screening for pathogenic genes related to hyperthyroidism in affected children was performed using the publicly available disease databases Malacards, MutationView, and Clinvar, and the functions and influences of the identified pathogenic genes were analyzed using statistical analysis and the gene enrichment approach.

**Results:**

Through GO enrichment analysis, it was found that the most significant gene ontology enrichment was the function “hormone activity” in terms of gene ontology molecular function. The corresponding mutated genes set that has common effects on hyperthyroidism in children included TG, CALCA, POMC, CGA, PTH, GHRL, FBN1, TRH, PRL, LEP, ADIPOQ, INS, GH1. The second most significant gene ontology enrichment was the function “response to peptide hormone” in terms of biological process. The corresponding mutated genes set that has common effects on hyperthyroidism in children included LRP6, TSC2, KANK1, COL1A1, CDKN1B, POMC, STAT1, MEN1, APC, GHRL, TSHR, GJB2, FBN1, GPT, LEP, ADIPOQ, INS, GH1. Through KEGG enrichment analysis, it was found that the most significant biological signaling pathway enrichment was the pathway “Thyroid hormone signaling pathway” function. The corresponding mutated genes set that has common effects on hyperthyroidism in children included NOTCH3, MYH7, TSC2, STAT1, MED13L, MAP2K2, SLCO1C1, SLC16A2, and THRB. The second most significant biological signaling pathway enrichment was the pathway “Hypertrophic cardiomyopathy” in terms of biological process. The corresponding mutated genes set that has common effects on hyperthyroidism in children included IGF1, CACNA1S, MYH7, IL6, TTN, CACNB2, LAMA2, and DMD.

**Conclusion:**

The mutated genes in children with hyperthyroidism were closely linked to function involved in “hormone activity” and “response to peptide hormone” in terms of the biological signaling pathway, and to the functional pathways involved in “Thyroid hormone signaling pathway” and “Hypertrophic cardiomyopathy” in terms of the biological signaling pathway.

## 1 Introduction

Hyperthyroidism is characterized by the excessive synthesis and release of thyroid hormones, leading to increased metabolism and sympathetic nervous system activity, resulting in symptoms such as palpitations, sweating, increased appetite, increased bowel movements, and weight loss. Its hallmark is an increase in the synthesis and secretion of thyroid hormones and thyroxine (T4) and triiodothyronine (T3) (1). According to an epidemiological survey report released in 2021, the overall incidence rate of hyperthyroidism in mainland China is approximately 1.78% (2). The overall incidence of hyperthyroidism in European children and adolescents is approximately 4.58 per 10,000 per year, with a higher prevalence in girls than boys (3). Children with hyperthyroidism may experience thyroid enlargement, increased appetite with weight loss, bulging eyes, irritability, excessive sweating, tachycardia, hyperactivity, and tremors (4). They may also have comorbid conditions such as attention deficit hyperactivity disorder (ADHD), regulatory disorders, anxiety, bipolar disorder, depression, and an increased risk of suicide. Children with hyperthyroidism’s physical and mental health can be severely affected (5). Therefore, early and accurate diagnosis, precise treatment, and improved prognosis of pediatric hyperthyroidism are of great clinical significance.

Currently, more than 100 genes have been found to be related to the development of hyperthyroidism (according to the Malacards database, MutationView database, and ClinVar database). Among these genes, those that have received more attention include (1): the thyroid stimulating hormone receptor (TSHR) gene. TSHR gene mutations are one of the causes of non-autoimmune hyperthyroidism (6, 7), and neonatal non-autoimmune hyperthyroidism is associated with the TSHR gene c.1856A>G (p.Asp619Gly) heterozygous mutation (8); (2) the cytotoxic T-lymphocyte associated protein 4 (CTLA4) gene. CTLA-4 gene mutations in the Chinese Han population are related to the development of hyperthyroidism (9–11); (3) the thyroid globulin (TG) gene. Specific TG SNP haplotypes are associated with hyperthyroidism (12), and hyperthyroidism patients with the TG gene E33SNP C/C genotype are more likely to relapse after discontinuing medication (13); (4) the GNAS complex locus (GNAS) gene. Gene mutations in GNAS are found in approximately 70% of patients with autonomous thyroid adenomas (14), and non-autoimmune hyperthyroidism is also associated with GNAS mutations (15); (5) the thyroid hormone receptor beta (THRB) gene. THRB gene mutations are related to a tendency towards thrombosis in patients with hyperthyroidism (16).

Research on gene mutations related to hyperthyroidism has mainly focused on individual genes, such as the study of TSHR gene mutations in congenital non-autoimmune hyperthyroidism (17–19), the impact of novel heterozygous TSHR gene mutations on hyperthyroidism (20), and special attention paid to its effects on hyperthyroidism (21). The role of BAFF gene mutations in the pathogenesis of hyperthyroidism is also studied (22). Other studies have explored the relationship between hyperthyroidism and other related diseases, such as the analysis of KCNJ18 gene mutations in hyperthyroidism associated with hypokalemic periodic paralysis (23), the study of BRAF gene mutations in hyperthyroidism associated with thyroid papillary carcinoma (24), and the study of UGT1A1 gene mutations in hyperthyroidism associated with liver failure (25).

Studies on gene mutations in children with hyperthyroidism have also mainly focused on single genes, including TSHR gene mutation analysis in children with hyperthyroidism (26, 27) and its relationship with congenital non-autoimmune hyperthyroidism in newborns (28), as well as functional gain-of-function mutations in the TSHR gene leading to increased function and thyroid growth (29). Additionally, there have been studies on gene sets, such as the age-related HLADRB1*03 allele in the Polish population (30) and gene sets significantly associated with early-onset hyperthyroidism, including BTNL2, NOTCH4, TNFAIP3, and CXCR4 (31). Other recent studies on pediatric hyperthyroidism have focused on prevention (32, 33) and treatment options (34–39), as well as research on related clinical symptoms (40, 41).

In summary, the genetic research on hyperthyroidism, especially pediatric hyperthyroidism, has mainly focused on single genes that affect the development of the disease. There are relatively few studies analyzing the expected effects of multiple mutated genes in hyperthyroidism. Therefore, more comprehensive understanding of the disease and help promote healthy development for children and adolescents, it is necessary to conduct more in-depth research and analysis on the functional implications of mutated genes in pediatric hyperthyroidism.

## 2 Materials and methods

### 2.1 WES data generation and gene processing

The cases were obtained from the Guangzhou Women and Children’s Medical Center. After approval from the Center’s Ethics Committee and obtaining informed consent from the guardians of the children with hyperthyroidism, peripheral blood samples were collected from 39 children with hyperthyroidism and DNA was extracted.

Whole-exome sequencing was performed on the DNA samples with GRCh38 as the reference genome. The main steps of Whole-exome sequencing include data filtering, data quality control, map to reference, mark duplicates, InDel realignment, base recalibration, the second data quality control, variant calling, variant filtering and variant annotation. GATK was used to detect the SNP and InDel. The reads map rate of the resulting data is 99%, indicating that the selection of the reference sequence is more accurate, and the average sequencing depth is 150X, which is enough for analysis, and 99% reaches 4X coverage.

The Whole-exome sequencing data were screened. This study obtained genes related to hyperthyroidism by searching public disease databases such as Malacards, MutationView, and Clinvar, comparing them with the mutated genes of 39 patients, and a total of 144 genes related to hyperthyroidism were screened. The specific genes are shown in **Table 1**.

**Table 1:** List of genes associated with hyperthyroidism of children studied.

No	Gene	No	Gene	No	Gene
1	ATAD3A	49	PCDH15	97	GYG2
2	RERE	50	CDH23	98	DMD
3	DDOST	51	INS	99	SLC16A2
4	PGM1	52	ANO5	100	MAGEC1
5	ACADM	53	F2	101	SRPK3
6	CACNA1S	54	MEN1	102	LMOD1
7	NLRP3	55	SYT12	103	POMC
8	TPO	56	DHCR7	104	GPT
9	DNMT3A	57	TECTA	105	FANCC
10	MSH2	58	VWF	106	FAM111A
11	MYO3B	59	CDKN1B	107	LRP6
12	TTN	60	GRIN2B	108	PRL
13	COL3A1	61	SLCO1C1	109	BMPR1A
14	STAT1	62	PFKM	110	STK11
15	CTLA4	63	CEP290	111	SDHA
16	CPS1	64	MED13L	112	EPS8
17	BARD1	65	ACADS	113	GRIN2A
18	TRNT1	66	GJB2	114	REN
19	GHRL	67	COG6	115	IL6
20	SCN10A	68	ATP7B	116	TSC2
21	TFG	69	MYH7	117	ACP5
22	CASR	70	CEP128	118	FKRP
23	TRH	71	TSHR	119	SERPINA7
24	GC	72	GOLGA6L6	120	PTH
25	ALB	73	SPG11	121	CAPN3
26	CCSER1	74	SLC24A5	122	KDM1A
27	TERT	75	FBN1	123	DCTN1
28	APC	76	STRA6	124	ADIPOQ
29	FBN2	77	GFER	125	NKAIN2
30	SPINK5	78	SRCAP	126	RAD51C
31	GRIA1	79	ANKRD11	127	ATM
32	KHDRBS2	80	ENO3	128	SHBG
33	COL12A1	81	BRCA1	129	LEP
34	CGA	82	COL1A1	130	CRP
35	LAMA2	83	MPO	131	POLG
36	PMS2	84	MAP2K2	132	SCO1
37	PLOD3	85	DNMT1	133	CEBPA
38	CFTR	86	NOTCH3	134	TNRC6B
39	KCNH2	87	RYR1	135	THRB
40	WRN	88	OPA3	136	NDUFV1
41	TG	89	NLRP12	137	PYGM
42	KANK1	90	GNAS	138	CALCA
43	GRHPR	91	SON	139	CDKN2A
44	PRUNE2	92	CHEK2	140	ELP3
45	LRSAM1	93	DEPDC5	141	NUBPL
46	COL5A1	94	SOX10	142	GH1
47	WDR37	95	PLA2G6	143	HSPB1
48	CACNB2	96	SHANK3	144	IGF1

### 2.2 Clinical data of the children studied

All the 39 children with hyperthyroidism in this study have corresponding clinical characteristics. The statistics of their clinical data are shown in the **Table 2** below. Female patients have more than male patients, Schoolagechild accounts for the largest proportion, 14 patients with family history of hyperthyroidism, 9 patients with abnormal liver function, the largest proportion of patients with second degree goiter, most of the patients’ TPOAb, TGAb, TG and TRAb indexes are abnormal.

**Table 2:** Clinical data of the children studied.

characteristic	value	count
Sex	Female	29
	male	10
age at diagnosis	Adolescence	5
	Preschool age child	4
	School age child	27
	Toddlers	3
family history of hyperthyroidism	Yes	14
	No	24
Abnormal liver function	Yes	9
	No	30
Goiter	1 degree	2
	2 degree	30
	3 degree	6
Exophthalmos	Yes	21
	No	17
TPOAb	normal	4
	abnormal	35
TGAb	normal	8
	abnormal	31
TG	normal	6
	abnormal	33
TRAb	normal	1
	abnormal	38

### 2.3 Functional enrichment analysis

The “clusterProfiler” R package was used for enrichment analysis of this paper. The basic principle of enrichment analysis is to compare the gene set to be analyzed from patients in section 1.1 with the reference gene set to determine which biological functions or pathways appear significantly more frequently in the gene set to be analyzed than in the reference gene set in the database, so as to find the biological processes, functions or pathways that have a significant impact on patients and the corresponding gene set.

### 2.4 Mutation data analysis and visualization

The “maftools” R package (42) was used to analyze a large amount of whole-exome sequencing data and visualize the data. First of all, we made a statistical analysis of the whole exon sequencing data set of screened genes: the distribution of high-frequency genes in each patient, the number of rare mutations compared with the 1000G_ALL database in the sample patients. Finally, the data distribution is displayed visually.

**Figure 1:**
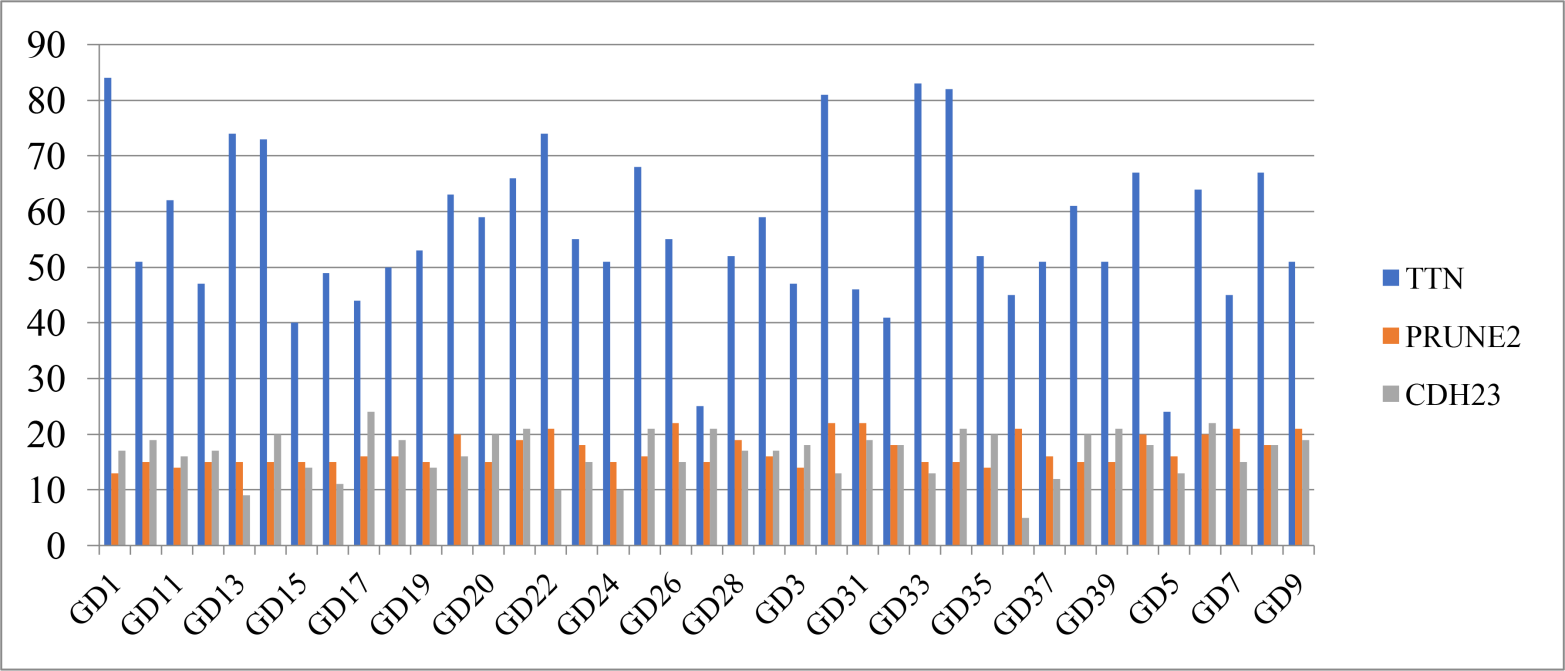
The distribution of the Top 3 high-frequency mutated genes on each sample.

**Table 3** shows the distribution of the top 10 high-frequency mutated genes on each sample. In this paper, the top 3 genes were selected for graphical display. **Figure 1** shows that the TTN gene has the highest mutation frequency in patient GD1, the PRUNE2 gene has the highest mutation frequency in patients GD30 and GD31, and the CDH23 gene has the highest mutation frequency in patient GD17.

**Table 3:** The distribution of the top 10 high-frequency mutated genes on each sample.

Sample	TTN	PRUNE2	CDH23	RYR1	SPINK5	SCN10A	VWF	NOTCH3	MYO3B	TG
**GD1**	84	13	17	14	5	4	5	9	7	8
**GD10**	51	15	19	18	14	5	13	8	8	8
**GD11**	62	14	16	7	17	10	6	8	8	5
**GD12**	47	15	17	19	16	12	6	9	7	7
**GD13**	74	15	9	15	18	6	9	8	10	10
**GD14**	73	15	20	14	14	10	4	9	10	9
**GD15**	40	15	14	6	16	9	12	8	5	6
**GD16**	49	15	11	17	16	11	12	9	11	7
**GD17**	44	16	24	18	14	10	10	9	11	5
**GD18**	50	16	19	16	14	3	6	9	6	8
**GD19**	53	15	14	21	16	6	6	9	10	8
**GD2**	63	20	16	13	16	9	8	1	7	8
**GD20**	59	15	20	8	4	14	11	9	5	7
**GD21**	66	19	21	5	16	4	11	7	8	8
**GD22**	74	21	10	16	5	9	12	9	10	4
**GD23**	55	18	15	18	3	3	4	8	10	10
**GD24**	51	15	10	6	15	10	5	8	8	6
**GD25**	68	16	21	7	3	10	4	9	12	14
**GD26**	55	22	15	16	4	9	9	10	8	9
**GD27**	25	15	21	20	16	9	7	9	8	10
**GD28**	52	19	17	17	17	11	4	7	8	10
**GD29**	59	16	17	5	14	9	6	9	10	9
**GD3**	47	14	18	9	14	9	8	7	9	6
**GD30**	81	22	13	8	14	11	10	8	7	12
**GD31**	46	22	19	9	3	8	11	8	6	10
**GD32**	41	18	18	18	14	8	9	7	10	6
**GD33**	83	15	13	19	15	12	9	9	4	9
**GD34**	82	15	21	17	16	9	7	8	7	7
**GD35**	52	14	20	18	14	9	6	8	10	7
**GD36**	45	21	5	9	16	6	12	7	5	11
**GD37**	51	16	12	18	17	14	6	9	7	6
**GD38**	61	15	20	17	14	4	6	8	6	4
**GD39**	51	15	21	18	16	10	9	8	8	9
**GD4**	67	20	18	3	17	9	7	9	9	6
**GD5**	24	16	13	8	14	14	12	4	5	10
**GD6**	64	20	22	9	14	3	4	7	10	10
**GD7**	45	21	15	20	3	9	13	7	9	9
**GD8**	67	18	18	8	5	9	6	8	8	6
**GD9**	51	21	19	18	14	8	11	9	5	3

**Figure 2:**
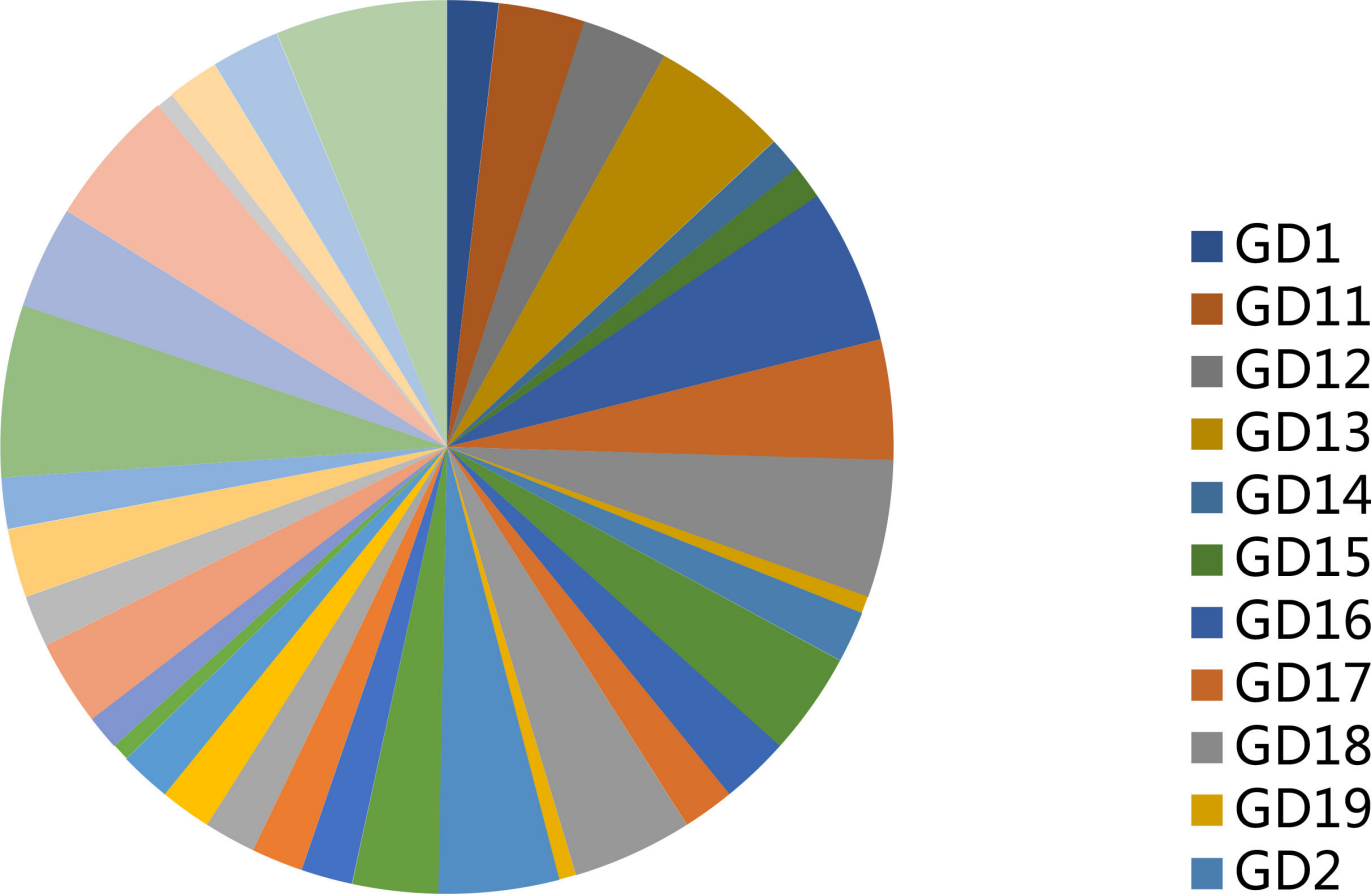
Rare variation proportion of each sample.

**Table 4** shows the rare variance of the top 10 high-frequency variant genes in each sample compared with the 1000G_ALL database, and **Figure 2** shows the proportion of rare variants corresponding to **Table 4** for each patient. It can be found that the proportion of rare variants in GD9 and GD39 is the largest.

**Table 4:** The rare variance of the top 10 high-frequency variant genes in each sample.

Sample	The number of rare mutations from top 10 high-frequency genes
**GD1**	3
**GD11**	5
**GD12**	5
**GD13**	8
**GD14**	2
**GD15**	2
**GD16**	9
**GD17**	7
**GD18**	8
**GD19**	1
**GD2**	3
**GD20**	6
**GD21**	4
**GD22**	3
**GD23**	7
**GD24**	1
**GD25**	7
**GD26**	5
**GD27**	3
**GD28**	3
**GD29**	3
**GD3**	3
**GD30**	3
**GD31**	1
**GD32**	2
**GD33**	5
**GD35**	3
**GD36**	4
**GD37**	3
**GD39**	10
**GD4**	6
**GD5**	8
**GD6**	1
**GD7**	3
**GD8**	4
**GD9**	10

**Figure 3:**
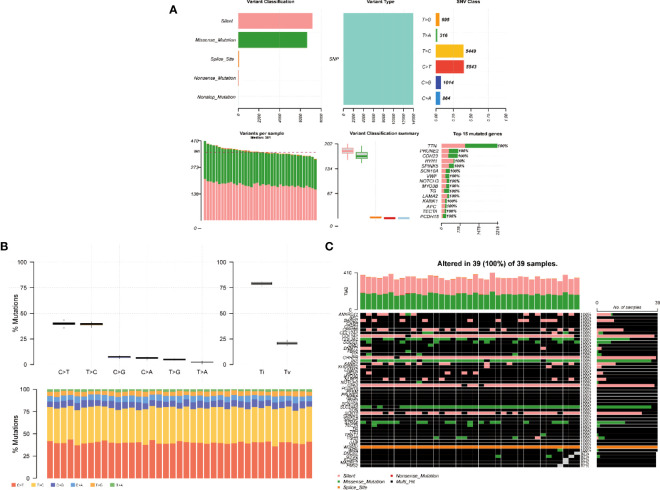
Somatic mutation profiles of hyperthyroidism in children. **(A)** Cohort summary plot of the mutation information among 39 cases of children with hyperthyroidism, the first row shows the statistical distribution of mutation classification, type, SNV class, the second row shows the distribution of mutation frequency and classification for each sample, and the last stacked statistic plot shows the top 15 most mutated genes. **(B)** Transition (Ti) and transversion(Tv) plot displays the distribution of SNVs in children with hyperthyroidism, and the stacked bar statistics show the distribution of SNVs in each child. **(C)** Landscape of mutation profiles shows mutation information of 39 cases of children with hyperthyroidism. The mutated genes are ordered by their mutation frequency.

A summary of the mutation information of hyperthyroidism in children was visualizing in **Figure 3**. The top 15 mutated genes of hyperthyroidism in children by percentage was shown in the figure, including TTN, PRUNE2, CDH23, RYR1, SPINK5, SCN10A, VWF, NOTCH3, MYO3B, TG, LAMA2, KANK1, APC, TECTA, PCDH15. The mutation rate of the 15 genes in 39 children with hyperthyroidism is 100% (**Figures 3A, B**). The synonymous mutation was the most common type of mutation in 39 children with hyperthyroidism, followed by missense mutations (**Figure 3A**). Among the SNV classes, T>C was the predominant type of SNVs detected (**Figure 3B**). Different mutated genes have different tendencies for mutation types in children with hyperthyroidism. Gene WDR37 tends to have Splice site mutations, Gene COL3A1, GRHPR, OPA3 and SOX10 tend to have Silent mutations, and Gene INS and SLC24A5 tend to have missense mutations (**Figure 3C**).

## 3 Results

### 3.1 GO enrichment analysis

With the ORA method, the screened 144 genes were used to perform a statistical analysis of the gene set using the GO database as a reference. The returned p-values were subjected to multiple testing FDR correction, deriving a significance index denoted as p.adjust. A lower p.adjust value indicates a higher significance level in the enrichment analysis.

**Figure 4:**
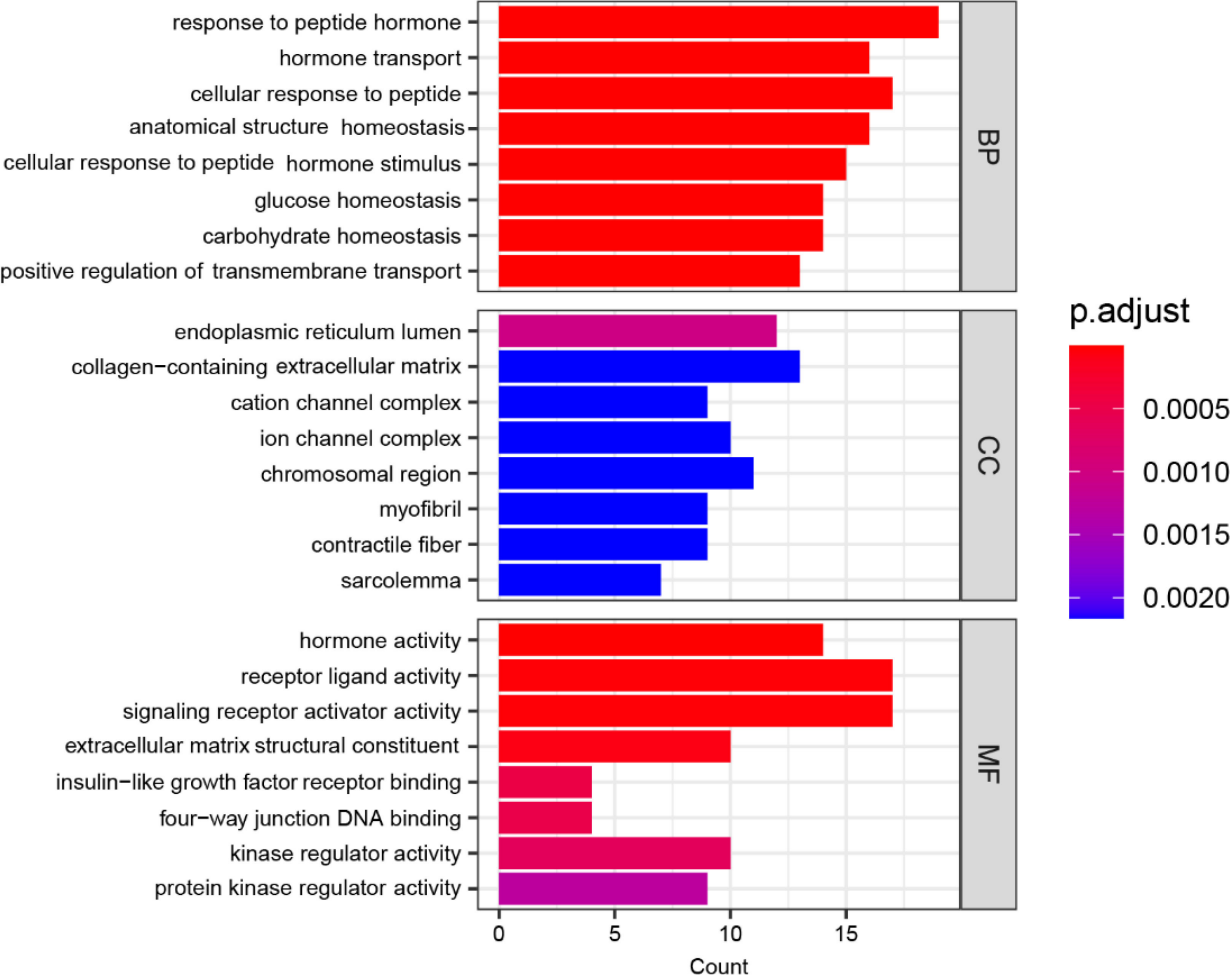
GO Enrichment analysis of hyperthyroidism in children.

**Figure 4** displays the functional enrichment of the 144 mutated genes in three dimensions of gene ontology. BP represents Biological Process: characterizing the biological processes in which genes are involved, such as cell differentiation and DNA replication; CC represents Cellular Component: describing where the gene product acts in which cellular component or gene product; MF represents Molecular Function: describing the activities of individual molecules in molecular biologies, such as protein kinase activity and insulin receptor activity. In **Figure 4**, the horizontal axis labeled “Count” denotes the quantity of genes in the enriched gene set, with longer bars indicating a more significant number of genes. The color coding represents the significance of the enrichment, with a redder color and a smaller p.adjust value indicating a higher significance level.

Based on the results presented in **Figure 4**, in terms of the Biological Process (BP) category, the most significant enrichment of mutated genes is in the “response to peptide hormone” function, followed by enrichment in “hormone transport”, “cellular response to peptide”, “anatomical structure homeostasis”, “cellular response to peptide hormone stimulus” and “glucose homeostasis”. In terms of the Cellular Component (CC) category, the most significant enrichment of mutated genes is in the “endoplasmic reticulum lumen” function, followed by enrichment in “collagen-containing extracellular matrix”, “cation channel complex”, “ion channel complex”, “chromosomal region” and “myofibril”. Similarly, within the Molecular Function (MF) category, the “hormone activity” function exhibits the most significant enrichment of mutated genes, followed by enrichments in functions “receptor ligand activity”, “signaling receptor activator activity”, “extracellular matrix structural constituent”, “insulin-like growth factor receptor binding” and “four-way junction DNA binding”.

The complete information of the top 8 significantly enriched biological processes (BP) terms are shown in **Table 5**, ranked by their level of significance based on the p.adjust values. The “Description” column describes the biological process associated with the enriched gene set, the “geneID” column lists all the genes in the gene set that has common effects, and the “Count” column indicates the number of genes in the enriched gene set.

**Table 5:** GO Enrichment results in terms of the BP category.

Description	p.adjust	geneID	Count
response to peptide hormone	9.02E-07	CPS1/LRP6/TSC2/KANK1/COL1A1/CDKN1B/POMC/STAT1/MEN1/APC/GHRL/TSHR/GJB2/FBN1/GPT/LEP/ADIPOQ/INS/GH1	19
hormone transport	1.43E-06	CFTR/CASR/POMC/SERPINA7/CGA/IL6/SLCO1C1/REN/SLC16A2/PFKM/GHRL/TRH/LEP/ADIPOQ/PLA2G6/INS	16
cellular response to peptide	1.43E-06	IGF1/CPS1/CASR/TSC2/KANK1/STAT1/MEN1/APC/GHRL/TSHR/GJB2/FBN1/GPT/LEP/ADIPOQ/INS/GH1	17
anatomical structure homeostasis	1.43E-06	CAPN3/ACP5/HSPB1/CDH23/CALCA/STK11/IL6/BARD1/PCDH15/PTH/PFKM/ALB/CACNB2/LAMA2/DMD/DCTN1	16
cellular response to peptide hormone stimulus	3.11E-06	CPS1/TSC2/KANK1/STAT1/MEN1/APC/GHRL/TSHR/GJB2/FBN1/GPT/LEP/ADIPOQ/INS/GH1	15
glucose homeostasis	4.26E-06	CFTR/CASR/POMC/STK11/MEN1/IL6/POLG/PFKM/GHRL/LEP/ADIPOQ/PLA2G6/CEBPA/INS	14
carbohydrate homeostasis	4.26E-06	CFTR/CASR/POMC/STK11/MEN1/IL6/POLG/PFKM/GHRL/LEP/ADIPOQ/PLA2G6/CEBPA/INS	14
positive regulation of transmembrane transport	4.26E-06	CFTR/IGF1/KCNH2/CAPN3/PTH/TERT/CACNB2/F2/ADIPOQ/DMD/SHANK3/INS/GH1	13

In the BP terms, the most significant enrichment of mutant genes was in the function of “response to peptide hormone” (p. adjust = 9.01×10-7) and the corresponding gene set that has common effects contained 19 genes: CPS1, LRP6, TSC2, KANK1, COL1A1, CDKN1B, POMC, STAT1, MEN1, APC, GHRL, TSHR, GJB2, FBN1, GPT, LEP, ADIPOQ, INS, GH1, followed by “hormone transport” (p. adjust = 1.43×10-6), with the corresponding gene set that has common effects containing 16 genes: CFTR, CASR, POMC, SERPINA7, CGA, IL6, SLCO1C1, REN, SLC16A2, PFKM, GHRL, TRH, LEP, ADIPOQ, PLA2G6, INS, “cellular response to peptide” (p. adjust = 1.43×10-6), with the corresponding gene set that has common effects containing 17 genes: IGF1, CPS1, CASR, TSC2, KANK1, STAT1, MEN1, APC, GHRL, TSHR, GJB2, FBN1, GPT, LEP, ADIPOQ, INS, GH1, “anatomical structure homeostasis” (p. adjust = 1.43×10-6), with the corresponding gene set that has common effects containing 16 genes: CAPN3, ACP5, HSPB1, CDH23, CALCA, STK11, IL6, BARD1, PCDH15, PTH, PFKM, ALB, CACNB2, LAMA2, DMD, DCTN1, “cellular response to peptide hormone stimulus” (p. adjust = 3.11×10-6), with the corresponding gene set that has common effects containing 15 genes: CPS1, TSC2, KANK1, STAT1, MEN1, APC, GHRL, TSHR, GJB2, FBN1, GPT, LEP, ADIPOQ, INS, GH1 and “glucose homeostasis” (p. adjust = 4.26×10-6), with the corresponding gene set that has common effects containing 14 genes: CFTR, CASR, POMC, STK11, MEN1, IL6, POLG, PFKM, GHRL, LEP, ADIPOQ, PLA2G6, CEBPA, INS.

The complete information of the top 8 significantly enriched cellular component (CC) terms are shown in **Table 6**, ranked by their level of significance based on the p.adjust values. The “Description” column provides a description of the cellular component associated with the enriched gene set, the “geneID” column lists all the genes in the gene set that has common effects and the “Count” column indicates the number of genes in the enriched gene set.

**Table 6:** GO enrichment results in terms of the CC category.

Description	p.adjust	geneID	Count
endoplasmic reticulum lumen	1.03E-03	PLOD3/COL1A1/COL12A1/COL5A1/MEN1/IL6/GHRL/ALB/FBN1/COL3A1/F2/INS	12
collagen-containing extracellular matrix	2.16E-03	PLOD3/COL1A1/TECTA/VWF/COL12A1/COL5A1/FBN2/FBN1/COL3A1/F2/ADIPOQ/LAMA2/GH1	13
cation channel complex	2.16E-03	KCNH2/CACNA1S/EPS8/GRIA1/CACNB2/GRIN2A/SCN10A/RYR1/GRIN2B	9
ion channel complex	2.16E-03	CFTR/KCNH2/CACNA1S/EPS8/GRIA1/CACNB2/GRIN2A/SCN10A/RYR1/GRIN2B	10
chromosomal region	2.16E-03	KDM1A/MSH2/DNMT3A/DNMT1/MEN1/APC/ATM/TERT/WRN/CHEK2/DCTN1	11
myofibril	2.16E-03	CACNA1S/MYH7/CAPN3/HSPB1/SCO1/TTN/LMOD1/RYR1/DMD	9
contractile fiber	2.16E-03	CACNA1S/MYH7/CAPN3/HSPB1/SCO1/TTN/LMOD1/RYR1/DMD	9
sarcolemma	2.16E-03	CACNA1S/CAPN3/CACNB2/FKRP/RYR1/LAMA2/DMD	7

In the CC terms, the most significant enrichment of mutant genes was in the function of “endoplasmic reticulum lumen” (p. adjust = 1.03×10-3) and the corresponding gene set that has common effects contained 12 genes: PLOD3, COL1A1, COL12A1, COL5A1, MEN1, IL6, GHRL, ALB, FBN1, COL3A1, F2, INS, followed by “collagen-containing extracellular matrix” (p. adjust = 2.16×10-3), with the corresponding gene set that has common effects containing 13 genes: PLOD3, COL1A1, TECTA, VWF, COL12A1, COL5A1, FBN2, FBN1, COL3A1, F2, ADIPOQ, LAMA2, GH1, “cation channel complex” (p. adjust = 2.16×10-3), with the corresponding gene set that has common effects containing 9 genes: KCNH2, CACNA1S, EPS8, GRIA1, CACNB2, GRIN2A, SCN10A, RYR1, GRIN2B, “ion channel complex” (p. adjust = 2.16×10-3), with the corresponding gene set that has common effects containing 10 genes: CFTR, KCNH2, CACNA1S, EPS8, GRIA1, CACNB2, GRIN2A, SCN10A, RYR1, GRIN2B, “chromosomal region” (p. adjust = 2.16×10-3), with the corresponding gene set that has common effects containing 11 genes: KDM1A, MSH2, DNMT3A, DNMT1, MEN1, APC, ATM, TERT, WRN, CHEK2, DCTN1 and “myofibril” (p. adjust = 2.16×10-3), with the corresponding gene set that has common effects containing 9 genes: CACNA1S, MYH7, CAPN3, HSPB1, SCO1, TTN, LMOD1, RYR1, DMD.

The complete information of the top 8 significantly enriched molecular function (MF) terms are shown in **Table 7**, ranked by significance level based on the p.adjust values. The “Description” column describes the molecular function associated with the enriched gene set, the “geneID” column lists all the genes in the gene set that has common effects and the “Count” column indicates the number of genes in the enriched gene set.

**Table 7:** GO Enrichment results in terms of the MF category.

Description	p.adjust	geneID	Count
hormone activity	1.63E-10	IGF1/TG/CALCA/POMC/CGA/PTH/GHRL/FBN1/TRH/PRL/LEP/ADIPOQ/INS/GH1	14
receptor ligand activity	2.55E-05	IGF1/TG/CALCA/POMC/GFER/CGA/IL6/PTH/GHRL/FBN1/TRH/PRL/LEP/F2/ADIPOQ/INS/GH1	17
signaling receptor activator activity	2.55E-05	IGF1/TG/CALCA/POMC/GFER/CGA/IL6/PTH/GHRL/FBN1/TRH/PRL/LEP/F2/ADIPOQ/INS/GH1	17
extracellular matrix structural constituent	7.41E-05	COL1A1/TECTA/VWF/COL12A1/COL5A1/FBN2/FBN1/COL3A1/ADIPOQ/LAMA2	10
insulin-like growth factor receptor binding	0.000454	IGF1/GNAS/REN/INS	4
four-way junction DNA binding	0.000491	MSH2/RAD51C/MEN1/WRN	4
kinase regulator activity	0.000644	LRP6/HSPB1/BMPR1A/CDKN1B/STK11/MAP2K2/ELP3/APC/CDKN2A/GHRL	10
protein kinase regulator activity	0.001278	HSPB1/BMPR1A/CDKN1B/STK11/MAP2K2/ELP3/APC/CDKN2A/GHRL	9

In the MF terms, the most significant enrichment of mutant genes was in the function of “hormone activity” (p. adjust = 1.63×10-10) and the corresponding gene set that has common effects contained 14 genes: IGF1, TG, CALCA, POMC, CGA, PTH, GHRL, FBN1, TRH, PRL, LEP, ADIPOQ, INS, GH1, followed by “receptor ligand activity” (p. adjust = 2.55×10-5), with the corresponding gene set that has common effects containing 17 genes: IGF1, TG, CALCA, POMC, GFER, CGA, IL6, PTH, GHRL, FBN1, TRH, PRL, LEP, F2, ADIPOQ, INS, GH1, “signaling receptor activator activity” (p. adjust = 2.55×10-5), with the corresponding gene set that has common effects containing 17 genes: IGF1, TG, CALCA, POMC, GFER, CGA, IL6, PTH, GHRL, FBN1, TRH, PRL, LEP, F2, ADIPOQ, INS, GH1, “extracellular matrix structural constituent” (p. adjust = 7.41×10-5), with the corresponding gene set that has common effects containing 10 genes: COL1A1, TECTA, VWF, COL12A1, COL5A1, FBN2, FBN1, COL3A1, ADIPOQ, LAMA2, “insulin-like growth factor receptor binding” (p. adjust = 4.54×10-4), with the corresponding gene set that has common effects containing 4 genes: IGF1, GNAS, REN, INS and “four-way junction DNA binding” (p. adjust = 4.91×10-4), with the corresponding gene set that has common effects containing 4 genes: MSH2, RAD51C, MEN1, WRN.

Overall, the functional significance of mutated genes in CC terms was relatively low in affected children and relatively high in BP terms. In all GO enrichments, the most significant functional enrichment of mutated genes in hyperthyroidism children was observed in “hormone activity” and “response to peptide hormone" functions.

### 3.2 KEGG enrichment analysis

With the ORA method, the 144 screened genes were used to perform a statistical analysis of the gene set using the KEGG biological signaling pathway database as a reference. The returned p-values were subjected to multiple testing FDR correction, deriving a significance index denoted as p.adjust. A lower p.adjust value indicates a higher significance level in the enrichment analysis.

**Figure 5:**
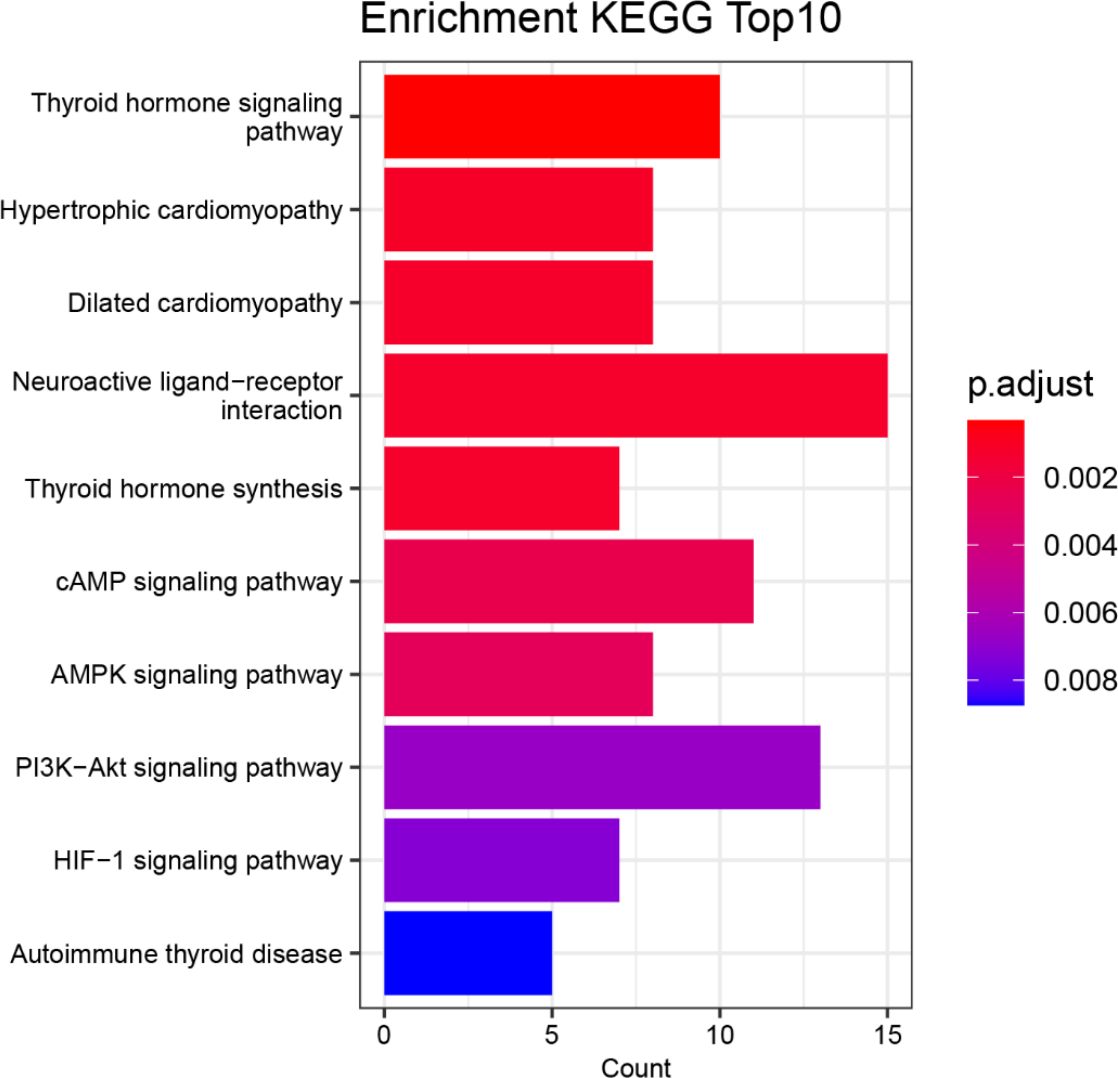
The top 15 pathways involved hyperthyroidism in children in by KEGG.

**Figure 5** displays the top 10 functional biological signaling pathways of the 144 mutant genes that were most significantly enriched. The horizontal axis labeled “Count” denotes the quantity of genes in the enriched gene set, with longer bars indicating a more significant number of genes. The color coding represents the significance of the enrichment, with a redder color and a smaller p.adjust value indicating a higher significance level.

Based on the results presented in **Figure 5**, the most significant enrichment of mutated genes is in the biological signaling pathway “Thyroid hormone signaling pathway” function, followed by enrichment in the biological signaling pathway “Hypertrophic cardiomyopathy”, “Dilated cardiomyopathy”, “Neuroactive ligand-receptor interaction”, “Thyroid hormone synthesis” and “cAMP signaling pathway”.

The complete information of the top 10 significantly functional biological signaling pathways are shown in **Table 8**, ranked by their significance level based on the p.adjust values. The “Description” column provides a description of the biological signaling pathways associated with the enriched gene set, the “geneID” column lists all the genes in the gene set that has common effects and the “Count” column indicates the number of genes in the enriched gene set.

**Table 8:** The results of KEGG enrichment.

Description	p.adjust	gene	count
Thyroid hormone signaling pathway	3.08E-04	NOTCH3, MYH7, TSC2, STAT1, MED13L, MAP2K2, SLCO1C1, SLC16A2, THRB, PFKM	10
Hypertrophic cardiomyopathy	1.10E-03	IGF1, CACNA1S, MYH7, IL6, TTN, CACNB2, LAMA2, DMD	8
Dilated cardiomyopathy	1.19E-03	IGF1, CACNA1S, GNAS, MYH7, TTN, CACNB2, LAMA2, DMD	8
Neuroactive ligand-receptor interaction	1.20E-03	CALCA, POMC, CGA, THRB, PTH, GRIA1, GHRL, TSHR, TRH, PRL, LEP, F2, GRIN2A, GH1, GRIN2B	15
Thyroid hormone synthesis	1.20E-03	TG, GNAS, TPO, SERPINA7, CGA, ALB, TSHR	7
cAMP signaling pathway	2.32E-03	CFTR, CACNA1S, GNAS, POMC, MAP2K2, CGA, GRIA1, GHRL, TSHR, GRIN2A, GRIN2B	11
AMPK signaling pathway	2.72E-03	CFTR, IGF1, TSC2, STK11, PFKM, LEP, ADIPOQ, INS	8
PI3K-Akt signaling pathway	6.70E-03	BRCA1, IGF1, TSC2, COL1A1, VWF, CDKN1B, STK11, MAP2K2, IL6, PRL, LAMA2, INS, GH1	13
HIF-1 signaling pathway	7.22E-03	IGF1, ENO3, CDKN1B, MAP2K2, IL6, PFKM, INS	7
Autoimmune thyroid disease	8.73E-03	TG, TPO, CGA, CTLA4, TSHR	5
Cushing syndrome	8.92E-03	CACNA1S, GNAS, CDKN1B, POMC, MAP2K2, MEN1, APC, CDKN2A	8
Human papillomavirus infection	8.92E-03	NOTCH3, GNAS, TSC2, COL1A1, VWF, CDKN1B, STAT1, MAP2K2, APC, ATM, TERT, LAMA2	12
Growth hormone synthesis secretion and action	9.00E-03	IGF1, CACNA1S, GNAS, STAT1, MAP2K2, GHRL, GH1	7
Long-term depression	1.11E-02	IGF1, GNAS, MAP2K2, GRIA1, RYR1	5
FoxO signaling pathway	1.32E-02	IGF1, CDKN1B, STK11, MAP2K2, IL6, ATM, INS	7
Endocrine resistance	1.46E-02	IGF1, NOTCH3, GNAS, CDKN1B, MAP2K2, CDKN2A	6
Amoebiasis	1.66E-02	GNAS, HSPB1, COL1A1, IL6, COL3A1, LAMA2	6
Homologous recombination	1.66E-02	BRCA1, RAD51C, BARD1, ATM	4
Prolactin signaling pathway	1.66E-02	STAT1, MAP2K2, CGA, PRL, INS	5
Platinum drug resistance	1.81E-02	BRCA1, MSH2, ATP7B, CDKN2A, ATM	5
p53 signaling pathway	1.81E-02	IGF1, TSC2, CDKN2A, ATM, CHEK2	5
Non-alcoholic fatty liver disease	2.27E-02	SDHA, IL6, NDUFV1, LEP, ADIPOQ, CEBPA, INS	7
Carbon metabolism	2.27E-02	CPS1, SDHA, ENO3, ACADS, PFKM, GPT	6
mTOR signaling pathway	2.27E-02	IGF1, LRP6, DEPDC5, TSC2, STK11, MAP2K2, INS	7
Ovarian steroidogenesis	2.78E-02	IGF1GNAS, CGA, INS	4
Pathways of neurodegeneration - multiple diseases	3.18E-02	LRP6, SDHA, CACNA1S, SPG11, MAP2K2, APC, IL6, GRIA1, NDUFV1, GRIN2A, RYR1, DCTN1, GRIN2B	13
Fanconi anemia pathway	3.18E-02	BRCA1, RAD51C, PMS2, FANCC	4
Longevity regulating pathway	3.25E-02	IGF1, TSC2, STK11, ADIPOQ, INS	5
Regulation of lipolysis in adipocytes	3.83E-02	GNAS, CGA, TSHR, INS	4
Circadian entrainment	4.37E-02	GNAS, GRIA1, GRIN2A, RYR1, GRIN2B	5
AGE-RAGE signaling pathway in diabetic complications	4.81E-02	COL1A1, CDKN1B, STAT1, IL6, COL3A1	5

The most significant biological signaling pathways enrichment of mutant genes was in the functional pathway of “Thyroid hormone signaling pathway” (p. adjust = 3.08×10-4) and the corresponding gene set that has common effects contained 10 genes: NOTCH3, MYH7, TSC2, STAT1, MED13L, MAP2K2, SLCO1C1, SLC16A2, THRB, PFKM, followed by the functional pathway “Hypertrophic cardiomyopathy” (p. adjust = 1.10×10-3), with the corresponding gene set that has common effects containing 8 genes: IGF1, CACNA1S, MYH7, IL6, TTN, CACNB2, LAMA2, DMD, the functional pathway “Dilated cardiomyopathy” (p. adjust = 1.19×10-3), with the corresponding gene set that has common effects containing 8 genes: IGF1, CACNA1S, GNAS, MYH7, TTN, CACNB2, LAMA2, DMD, the functional pathway “Neuroactive ligand-receptor interaction” (p. adjust = 1.20×10-3), with the corresponding gene set that has common effects containing 15 genes: CALCA,POMC,CGA,THRB,PTH, GRIA1, GHRL, TSHR, TRH, PRL, LEP, F2, GRIN2A, GH1, GRIN2B, the functional pathway “Thyroid hormone synthesis” (p. adjust = 1.20×10-3), with the corresponding gene set that has common effects containing 7 genes: TG, GNAS, TPO, SERPINA7, CGA, ALB, TSHR and the functional pathway “cAMP signaling pathway” (p. adjust = 2.32×10-3), with the corresponding gene set that has common effects containing 11 genes: CFTR, CACNA1S, GNAS, POMC, MAP2K2, CGA, GRIA1, GHRL, TSHR, GRIN2A, GRIN2B.

## 4 Discussion

The collective action of multiple genes and various genetic factors contribute to the development of hyperthyroidism. The improvement of bioinformatics has provided new approaches and tools for studying diseases such as hyperthyroidism. In this study, we processed and statistically analyzed the whole-exome sequencing data of 39 children with hyperthyroidism. We used bioinformatics tools to perform GO enrichment analysis and KEGG enrichment analysis to obtain the significant functional gene sets associated with hyperthyroidism in children, and to help understand the pathogenesis of hyperthyroidism in polygenic diseases and the common effects of its mutated genes on different functions.

Through the mutation data visualization, we found that among the 144 mutated genes in 39 children with hyperthyroidism, the top 15 most mutated genes were TTN, PRUNE2, CDH23, RYR1, SPINK5, SCN10A, VWF, NOTCH3, MYO3B, TG, LAMA2, KANK1, APC, TECTA, PCDH15, and the top 45 genes with high mutation frequencies had mutations in each patient. In addition, Gene WDR37 tends to have Splice site mutations, Gene COL3A1, GRHPR, OPA3 and SOX10 tend to have Silent mutations, and Gene INS and SLC24A5 tend to have missense mutations. Among the mutation classifications, Synonymous mutation was the most common mutation classification in children with hyperthyroidism, followed by missense mutations. Regarding single nucleotide variations, T>C had the highest mutation frequency.

Through GO enrichment analysis, we found that in terms of the biological process (BP), the enriched functional gene set associated with hyperthyroidism in children was most significant for function “response to peptide hormone” with the p.adjust value of 9.01×10-7 and the corresponding gene set with mutations included 19 genes: CPS1, LRP6, TSC2, KANK1, COL1A1, CDKN1B, POMC, STAT1, MEN1, APC, GHRL, TSHR, GJB2, FBN1, GPT, LEP, ADIPOQ, INS, GH1. In terms of the cellular component (CC), the enriched functional gene set associated with hyperthyroidism in children was most significant for the function “endoplasmic reticulum lumen”, its p.adjust value is 1.03×10-3. The corresponding gene set with mutations included 12 genes: PLOD3, COL1A1, COL12A1, COL5A1, MEN1, IL6, GHRL, ALB, FBN1, COL3A1, F2, INS. In terms of the molecular function (MF), the enriched functional gene set associated with hyperthyroidism in children was most significant for function “hormone activity” with the p.adjust value of 1.63×10-10 and the corresponding gene set with mutations included 14 genes: IGF1, TG, CALCA, POMC, CGA, PTH, GHRL, FBN1, TRH, PRL, LEP, ADIPOQ, INS, GH1. In all GO enrichments, the significance of function “hormone activity” and “response to peptide hormone” was the highest. From the overall view of GO enrichment, the significance of mutated genes in children with hyperthyroidism was relatively low in the function of cell sublocalization level, and relatively high in the biological process level. This indicates that the mutated gene may affect more biological processes and molecular functions of children, such as internal circulation, hormone secretion, and the cell structure is not affected. From the gene ontology level, the mutant genes in hyperthyroidism children have the highest significance in the function of “hormone activity” and “response to peptide hormones”. In the GO database, “hormone activity” includes corticotropin releasing hormone activity, thyrotropin releasing hormone activity and other series of hormone activities. Studies have shown that corticotropin releasing hormone can affect the degree of hyperthyroidism (43). Thyrotropin releasing hormone is one of the hormones that regulate the level of serum thyroid hormone in a certain range, so the mutated gene can affect the incidence of hyperthyroidism by affecting the secretion of these hormones (44). Mutated genes also affect hyperthyroidism by influencing biological processes called “response to peptide hormones.”

Through KEGG enrichment, the enriched functional gene set associated with hyperthyroidism in children was most significant for function “Thyroid hormone signaling pathway” in the signal pathway with the p.adjust value of 3.08×10-4. The corresponding gene set with mutations included 10 genes: NOTCH3, MYH7, TSC2, STAT1, MED13L, MAP2K2, SLCO1C1, SLC16A2, THRB, PFKM. “Hypertrophic cardiomyopathy” in the signal pathway was followed with the p.adjust value of 1.10×10-3 and the corresponding collective action gene set with mutations included 8 genes: IGF1, CACNA1S, MYH7, IL6, TTN, CACNB2, LAMA2, DMD. Therefore, from a pathway perspective, the mutated gene affects the thyroid hormone-related interactions of biomolecules and chemical reactions in children leading to their disease and increases the risk of hyperthyroid hypertrophic cardiomyopathy in children.

## Data availability statement

The original contributions presented in the study are included in the article, further inquiries can be directed to the corresponding author.

## Ethics statement

The studies involving human participants were reviewed and approved by Guangzhou Women and Children’s Medical Center. Written informed consent to participate in this study was provided by the participants’ legal guardian/next of kin. Written informed consent was obtained from the individual(s), and minor(s)’ legal guardian/next of kin, for the publication of any potentially identifiable images or data included in this article.

## Author contributions

Conceptualization ideas: XM, LT. Data curation: XM, LT, HL,YH. Investigation: XM, WZ. Statistical analysis and interpretation: XM, LT, HL, LL,YH. Methodology: XM, LT, HL, WZ, LL, AH. Resources: XM, HL, WZ, LL. Supervision: XM. Project administration: XM,YH. Visualization: LT, HL, AH. Writing – original draft: XM, LT, HL,YH. Writing—review and editing: XM, LT, WZ. All authors read, contributed to the research design, and approved the final manuscript.
